# Immune Repertoire Sequencing Using Molecular Identifiers Enables Accurate Clonality Discovery and Clone Size Quantification

**DOI:** 10.3389/fimmu.2018.00033

**Published:** 2018-02-05

**Authors:** Ke-Yue Ma, Chenfeng He, Ben S. Wendel, Chad M. Williams, Jun Xiao, Hui Yang, Ning Jiang

**Affiliations:** ^1^Institute for Cellular and Molecular Biology, The University of Texas at Austin, Austin, TX, United States; ^2^Department of Biomedical Engineering, Cockrell School of Engineering, The University of Texas at Austin, Austin, TX, United States; ^3^McKetta Department of Chemical Engineering, Cockrell School of Engineering, The University of Texas at Austin, Austin, TX, United States; ^4^ImmuDX, LLC, Austin, TX, United States; ^5^School of Life Sciences, Northwestern Polytechnical University, Xi’an, Shaanxi, China; ^6^Research Center of Special Environmental Biomechanics & Medical Engineering, Xi’an, Shaanxi, China

**Keywords:** MID clustering-based IR-Seq TCR repertoire sequencing, molecular identifiers, sub-clustering, naïve T cells, CMV-specific T cells

## Abstract

Unique molecular identifiers (MIDs) have been demonstrated to effectively improve immune repertoire sequencing (IR-seq) accuracy, especially to identify somatic hypermutations in antibody repertoire sequencing. However, evaluating the sensitivity to detect rare T cells and the degree of clonal expansion in IR-seq has been difficult due to the lack of knowledge of T cell receptor (TCR) RNA molecule copy number and a generalized approach to estimate T cell clone size from TCR RNA molecule quantification. This limited the application of TCR repertoire sequencing (TCR-seq) in clinical settings, such as detecting minimal residual disease in lymphoid malignancies after treatment, evaluating effectiveness of vaccination and assessing degree of infection. Here, we describe using an MID Clustering-based IR-Seq (MIDCIRS) method to quantitatively study TCR RNA molecule copy number and clonality in T cells. First, we demonstrated the necessity of performing MID sub-clustering to eliminate erroneous sequences. Further, we showed that MIDCIRS enables a sensitive detection of a single cell in as many as one million naïve T cells and an accurate estimation of the degree of T cell clonal expression. The demonstrated accuracy, sensitivity, and wide dynamic range of MIDCIRS TCR-seq provide foundations for future applications in both basic research and clinical settings.

## Introduction

Immune repertoire sequencing (IR-seq) has become a useful tool to quantify the composition of B or T cell antigen receptor repertoires in basic research, such as vaccination (1–3), immune repertoire development (4–9), and lymphocyte lineage tracking (2, 9), as well as in various clinical settings, such as minimal residual disease (MRD) monitoring (10), hematopoietic stem cell transplant recovery monitoring (11), and cancer patient prognosis (12, 13). However, early IR-seq experiments suffered from high PCR and sequencing errors that limited their ability to perform accurate repertoire diversity and abundance quantification. This bottleneck also limits the sensitivity of many IR-seq-based assays, such as MRD monitoring. Recently, we and others introduced molecular identifiers (MIDs) to IR-seq and DNA/RNA sequencing to reduce errors by tracking each RNA molecule through PCR and sequencing. This approach has significantly improved the accuracy of repertoire profiling (9, 14–19), especially to distinguish antibody somatic hypermutations from PCR and sequencing errors. However, several challenges remain regarding how to use MIDs correctly and how to use MIDs for cell clone size estimate. First, erroneous MIDs resulting from PCR or sequencing errors make accurate MID counting difficult. Second, there is a lack of general guidelines of required sequencing depth to saturate MID counts. Third, how to use RNA molecular counting to estimate T cell clone size has yet to be established.

These challenges become roadblocks to accurately quantify T cell receptor (TCR) or BCR RNA molecule copy number, which is important in estimating clonal expansion and identifying rare clones. Robins et al. developed QuanTILfy to attempt to address this problem by counting TILs and assessing T cell clonality in tissue samples through droplet digital PCR (dPCR) of rearranged TCRβ loci (20). However, by partitioning TCR Vβ into eight non-overlapping subgroups, this method lacks the sensitivity to identify unique CDR3 of each clonality, not to mention rare clones. Therefore, a more comprehensive method to quantify TCR or antibody transcripts with high sensitivity while retaining accurate clonal diversity is needed for both standardizing basic IR-seq studies and applying it in clinical decision-making, such as detecting MRD in lymphoid malignancies after treatment, evaluating effectiveness of vaccination, and assessing degree of infection.

We recently developed a more generalized approach with reduced MID length to identify each individual RNA molecule using a sequence-similarity-based clustering method to separate sequencing reads into sub-clusters within a group of sequencing reads that have the same MID. We applied this MID Clustering-based IR-Seq (MIDCIRS) to study age-related antibody repertoire development and diversification during acute malaria (9). In this study, we applied MIDCIRS to TCR [MIDCIRS TCR repertoire sequencing (TCR-seq)] and used CD8^+^ T cells as a test bed to build a model to count TCR RNA molecule copy number based on input cell numbers, percentage of RNA input, and sequencing depth. We also demonstrated a significant improvement in detection sensitivity. A previous study using a different repertoire sequencing methodology reported the capacity to resolve one in 10,000 cells (21). With MIDCIRS TCR-seq, we were able to detect one unique T cell clone in 1,000,000 T cells. In addition, we applied MIDCIRS TCR-seq to examine T cell clonal expansion in CMV infection and showed that sensitive and accurate quantification of the TCR RNA molecule copy number is essential to quantify a single-cell’s worth of TCR transcripts and to assess the degree of clonal expansion. In summary, we showed the significance of the sub-clustering step of MIDCIRS in preventing false MID group generation, which enabled highly accurate clonal type discovery. This study provides a framework for leveraging the sensitivity and accuracy of molecular barcoded IR-seq in MRD detection and assessing clonal expansion in infection and vaccination.

## Materials and Methods

### Naïve CD8^+^ T Cell Sorting

Human leukocyte reduction system chambers were obtained from de-identified donors at We Are Blood (Austin, TX, USA) with strict adherence to guidelines from the Institutional Review Board of the University of Texas at Austin. CD8^+^ T cell enrichment was done following the protocol described previously (22) using RosetteSep CD8^+^ T Cell Enrichment Cocktail (STEMCELL) together with Ficoll-Paque (GE Healthcare). Then, RBCs were lysed using ACK Lysing Buffer (Lonza). After washing in phosphate-buffered saline with fetal bovine serum, the cell mixture was passed through a cell strainer (Corning) and ready for use. Naïve CD8^+^ T cells were FACS-sorted into RLT Plus buffer (Qiagen) supplemented with 1% β-mercaptoethanol (Sigma) based on the phenotype of CD8^+^CD4^-^CCR7^+^CD45RA^+^ using BD FACSAria II cell sorter.

### CMV CD8^+^ T Cell Enrichment and Sorting

CMVpp65:482-490 (NLVPMVATV) was used to prepare streptamers as previously described (23). Miltenyi anti-phycoerythrin microbeads and magnetic column were used to bind and enrich CMVpp65-specific T cells (22). The flow-through was collected for background staining. The enriched fraction was eluted off the column and washed into cell buffer. The following antibody panel was used to stain both the enriched and flow-through fractions: CD4, CD14, CD16, CD19, CD32, and CD56 (BioLegend) as a dump channel to stain residual non-CD8 T cells, and CD45RA, CCR7, CD27, and IL7R (BioLegend). 7-aminoactinomycin D was used as a viability marker. Dump^−^Streptmer^+^CD45RA^+^CCR7^−^CD27^−^IL7R^lo^ live T cells were sorted into RLT Plus buffer supplemented with 1% β-mercaptoethanol using BD FACSAria II cell sorter.

### Bulk TCR Library Generation and Sequencing

Total RNA was purified using All Prep DNA/RNA kit (Qiagen) following the manufacturer’s protocol. Library preparation and QC were similar to protocols described previously (9) using TCR primers (Table S5 in Supplementary Material). Reads of the same library from all runs were combined and analyzed.

### dPCR of TCR

Total RNA purified from sorted CD8^+^ T cells and cultured CMV-specific CD8^+^ T cell lines were reverse transcribed with polyT primers (Table S5 in Supplementary Material) using Superscript III in 20 µl reaction following the manufacturer’s protocol. 2 µl of cDNA was subsequently used on QuantStudio 3D dPCR system following manufacturer’s protocol.

### Preliminary Read Processing

We followed the similar procedure as described previously to generate consensus sequences (9). First, only reads that have exact TCR constant sequences were kept for further analysis. These reads were then cut to 150 nt starting from constant region to eliminate high error-prone region at the end of reads. These preprocessed reads were split into MID groups according to 12-nt barcodes.

### MID Sub-Cluster Generating and Filtering

For each MID group, a quality threshold clustering was used to group reads derived from a common ancestor RNA molecule and separate reads derived from distinct RNAs as previously described (9). Briefly, a Levenshtein distance of 15% of the read length was used as the threshold (9). For each subgroup, a consensus sequence was built based on the average nucleotide at each position, weighted by the quality score. In the case that there were only two reads in an MID subgroup, we only considered them useful reads if both were identical. Each MID subgroup is equivalent to an RNA molecule. Next, we merged all of the identical consensus to form unique consensus sequences. Further, we applied filtering of unique consensus sequences after sub-cluster generation by (a) removing non-functional TCR sequences and (b) removing sequences with lower MID counts that are one Levenshtein distance away from the other. Then, for each unique consensus sequence, we removed MID sub-clusters if their reads are less than 20% of maximum read count based on the fitting of two negative binomial distribution (Figure S5 in Supplementary Material). Scripts for this section can be downloaded at https://github.com/utjianglab/MIDCIRS.

### Theoretical Percentage of MIDs That Need Sub-Clustering

We modeled the process of MID labeling as a Poisson distribution. Given the total number of MIDs being *M* and the number of target molecules being *N*, the probability that a unique MID will occur *k* time(s) is:

(1) $$P_{k}=\frac{{\left(\frac{N}{M}\right)}^{k}}{k!}\times e^{-\frac{N}{M}}.$$

Thus, *P*_0_ and *P*_1_ are the probability that a MID will be tagged 0 and 1 time, respectively, and the percentage of MIDs that need sub-clustering, *F*(*k* > 1), is given by:

(2) $$F\left(k>1\right)=\frac{\left[1-e^{-\frac{N}{M}}-\frac{N}{M}\times e^{-\frac{N}{M}}\right]}{1-e^{-\frac{N}{M}}}.$$

**Figure 1:**
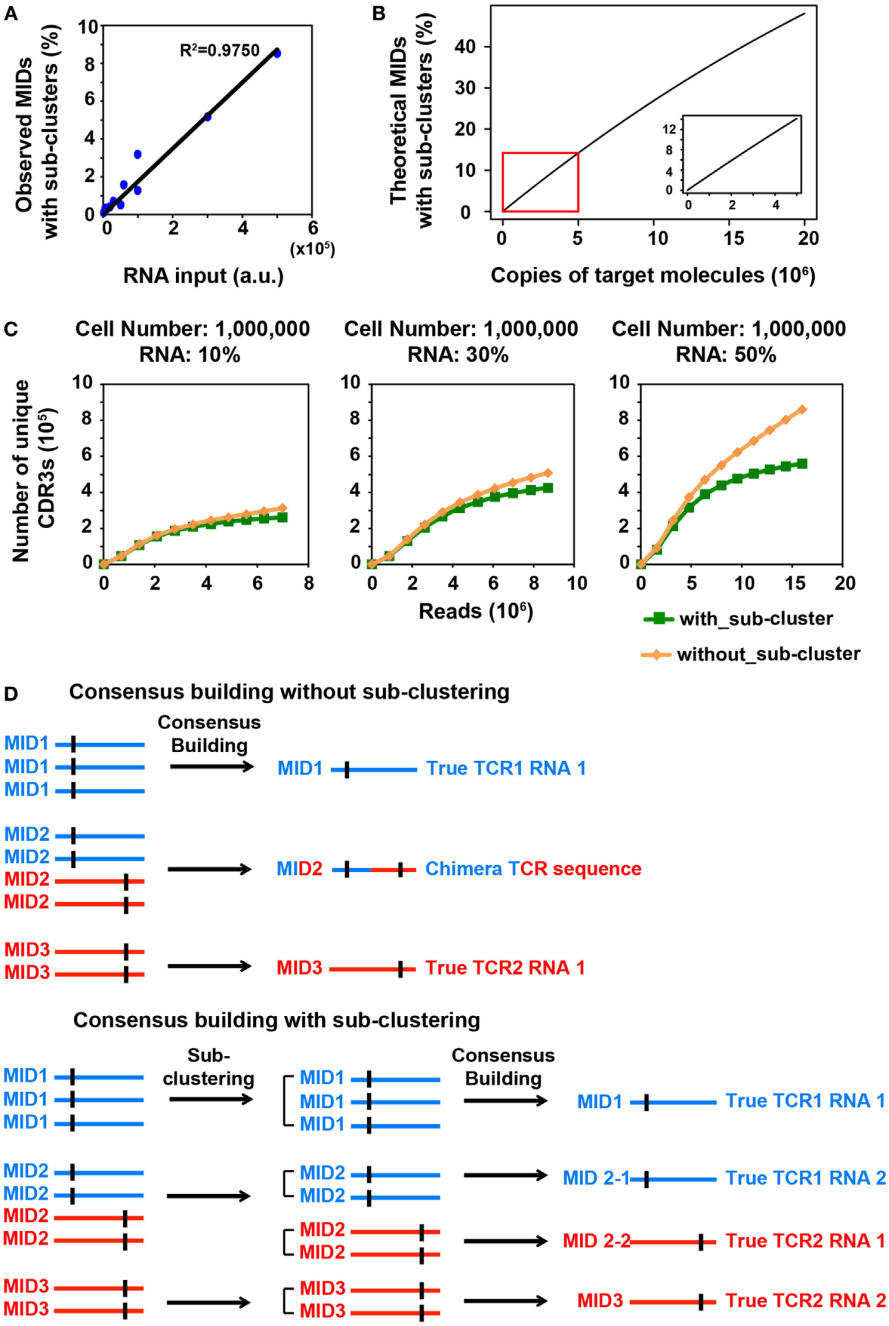
MID Clustering-based IR-Seq improves accuracy of T cell receptor (TCR) diversity estimation with sub-clustering. **(A)** The percentage of observed molecular identifiers (MIDs) containing sub-clusters is linearly dependent on RNA input, which is defined as cell number multiplied by percentage of RNA (e.g., 20,000 cells with 10%RNA is equivalent to 2,000 RNA input). Line represents linear regression fit, *F*-test on the slope, *p* < 10^−9^. **(B)** The theoretical percentage of MIDs with sub-clusters is approximately linearly dependent on copies of target molecules when copies of target molecules are less than 5,000,000 (bottom right insert). The theoretical percentage of MIDs with sub-clusters was calculated by Eq. 2 in Section “Materials and Methods.” **(C)** Rarefaction curve of unique complementarity-determining regions 3 (CDR3s) with or without sub-clustering. Number of unique CDR3s in three libraries made with three different RNA inputs from sorted one million naïve CD8^+^ T cells are shown here. Data from other cell inputs are in Figure S2 in Supplementary Material. **(D)** Illustration of consensus TCR sequence building without (top) and with (bottom) sub-clustering. Top: without sub-clustering, chimera sequences are generated when different TCR RNA molecules are tagged with the same MID; bottom: TCR RNA molecules that are tagged with same MID are sub-clustered to reveal truly represented TCR sequences. Short vertical black lines indicate nucleotide differences between two TCR sequences.

With over 16 million MID combinations from 12 random nucleotides, when the number of target molecules, *N* is less than 5,000,000, Eq. 2 is an approximate linear function (Figure 1B).

### Diversity Coverage and RNA Copy Number Simulation

The estimation of diversity will be affected by the initial RNA input (percentage of initial RNA used to construct the sequencing library). We used a statistical model to estimate the diversity coverage for the naïve T cells we sorted based on RNA sampling depth.

For *N* observed RNA molecules, there are *K* different RNA clones. The RNA molecule copy number of each clone is *m*_i_ (*i*∈(1,*K*)), whose sum equals *N*. After fitting the data, *m*_i_ follows a power law distribution (Figure S9 in Supplementary Material):

(3) $$m_{i}=m\times x_{i}$$

(4) $$f\left(x_{i}\right)=\left(\alpha -1\right)x_{i}^{-\alpha },(\alpha >1)$$

where, *m* is the RNA molecule copy number per cell, which is a constant across all T cells (see Figure 3C). *x*_i_ represents the cell numbers of each clone, which follows a power law distribution (24), and the parameter α was fitted with an algorithm combining maximum-likelihood fitting and goodness-of-fit test based on Kolmogorov–Smirnov statistic (25) “fit_power_law” function in R package igraph was applied (26).

Specifically, we fitted the RNA molecule distribution (Figure S9 in Supplementary Material) with Eq. 5:

(5) $$f\left(m_{i}\right)=\left(\frac{\alpha -1}{m_{\text{min}}}\right){\left(\frac{m_{i}}{m_{\text{min}}}\right)}^{-\alpha },(\alpha >1).$$

Since “*m*” is a constant (see Figure 3C), the alpha in Eqs 4 and 5 should be equal. We fitted across all libraries on log–log scale, and the average slope was taken as α in the above model.

When we sample *n* RNA molecules from this population, the expected detected diversity, *E*(D), can be calculated as the following:

(6) $$E\left(D|m,x_{i}\right)=K-\frac{\sum_{i=1}^{K}\left(\begin{array}{c} N-m\times x_{i}\\ n \end{array}\right)}{\left(\begin{array}{c} N\\ n \end{array}\right)},x_{i}=\left(x_{1},x_{2},\ldots ,x_{K}\right).$$

And *x*_i_ can be sampled from the fitted power law distribution.

Then, the percentage of the RNA diversity coverage, *P*(D), can be estimated as:

(7) $$P(D|m,x_{i})=\frac{E(D|m,x_{i})}{K}.$$

We scaled the diversity coverage of unique CDR3s to the estimated diversity coverage with 90% RNA input, *D*_obs_. We then used Eq. 8 to get estimated *m*:

(8) $$\underset{m}{\text{min}}\sum_{i}{\left(P\left(D_{i}|m,x_{i}\right)-D_{\text{obs}}\right)}^{2},m\in \left\{1,2,\ldots \right\}.$$

### Statistical Analysis

Mann–Whitney *U* test was used to calculate the significance of copy number difference between pairs in naïve, effector, effector memory, and central memory CD8^+^ T cells and *p* values was adjusted with Benjamini–Hochberg procedure. Adjusted *p*-value that was less than 0.05 was considered significant.

## Results

### MIDCIRS Sub-Clustering Improves Repertoire Diversity Estimation Accuracy

Molecular identifiers have been adopted in IR-seq and DNA/RNA sequencing to reduce error rate. However, during reverse transcription, multiple transcripts could stochastically be tagged with same MID. Previous strategies relied on increasing the length of MID to reduce the probability of non-unique MID tagging when the total RNA molecule copy number was either unknown or very large (27). However, longer MID length could reduce the efficiency of reverse transcription (28, 29). Thus, we developed a more generalized approach (MIDCIRS) with reduced MID length. A sequence-similarity-based clustering method was implemented in MIDCIRS to separate sequencing reads into sub-clusters within a group of sequencing reads that have the same MID (9). Here, we developed metrics to validate the accuracy of this sub-clustering method. In addition, we demonstrated the robust ability of MIDCIRS to faithfully represent the diversity and abundance of the TCR repertoire using a large range of RNA inputs.

We reasoned that in order to comprehensively quantify the overall diversity, a large portion of its RNA must be sampled. However, this will inevitably increase the number of TCR transcripts that need to be tagged with MIDs, which increases the portion of MIDs tagging multiple TCR transcripts. We sought to closely examine the relationship between RNA input and multiple TCR RNA tagging by the same MID. The process of MID labeling can be modeled as a Poisson distribution (see Materials and Methods). The percentage of MIDs with sub-clusters follows an approximate linear trend when the copies of target RNA molecules are less than 5,000,000 (Figure 1B). To experimentally validate this, we applied MIDCIRS TCR-seq on a range of sorted naïve CD8^+^ T cells (from 20,000 to 1 million) with three different RNA inputs (10, 30, and 50%) (Table S1 in Supplementary Material). We have previously used control template sequences and evaluated the clustering threshold that would separate TCR RNA molecules accidentally tagged with the same MID, which is 15% of the sequence length (9). As expected, we found that the observed percentage of MIDs that need sub-clustering is approximately linear with respect to copies of target RNA molecules used in this study (Figure 1A). With the highest amount of RNA molecules used in this study, approximately 8.5% of MIDs require further clustering, while previous method treated these sequences as ambiguous (17). Thus, MIDCIRS sub-clustering significantly improves repertoire diversity coverage.

To evaluate the accuracy of the sub-clustering step by an alternative means, we examined the TCR sequence lengths within MIDs that contain sub-clusters. We reasoned that if indeed each TCR RNA molecule was tagged with a unique MID, then the lengths of CDR3 for all reads would be identical under each MID. However, we showed that of the 8.5% of MIDs that contain sub-clusters, about 87% of MIDs contain TCR sequencing reads of different CDR3 lengths while only 13% have the same length for one million naïve CD8^+^ T cells (50% RNA input). After performing sub-clustering, over 97% of sub-clusters have a uniform length (Figure S1 in Supplementary Material), demonstrating the accuracy of sub-clustering step in MIDCIRS.

More importantly, to our surprise, we found that, without performing sub-clustering, the number of unique consensus sequences (unique CDR3 sequences) was overestimated, especially in samples with one million cells (Figure 1C; Figure S2 in Supplementary Material). This is because chimera sequences were generated in the consensus building step for two scenarios. In one scenario, multiple true TCR sequences could be tagged with the same MID and quality score weighted consensus building will generate chimera sequences (Figure 1D; Figure S3A in Supplementary Material). In the second scenario, PCR or sequencing errors on MIDs group multiple singletons (MIDs that contain only one read) under the new MID. If sub-clustering is applied, then these singletons will be separated and discarded under the singleton category. However, without sub-clustering, these singletons will be forced to generate a chimera sequence (Figure S3B in Supplementary Material). Taking together, these chimera sequences cause overestimation of the total TCR diversity. The percentage of chimera sequences can be as high as 47% (Table S1 in Supplementary Material). Thus, compared with previous IR-seq with MID method (17), MIDCIRS not only can increase diversity coverage of CDR3 but improve the accuracy of diversity estimation.

### MID Read-Distribution-Based Barcode Correction Improves Accuracy and Sensitivity of Counting TCR Transcripts

**Figure 2:**
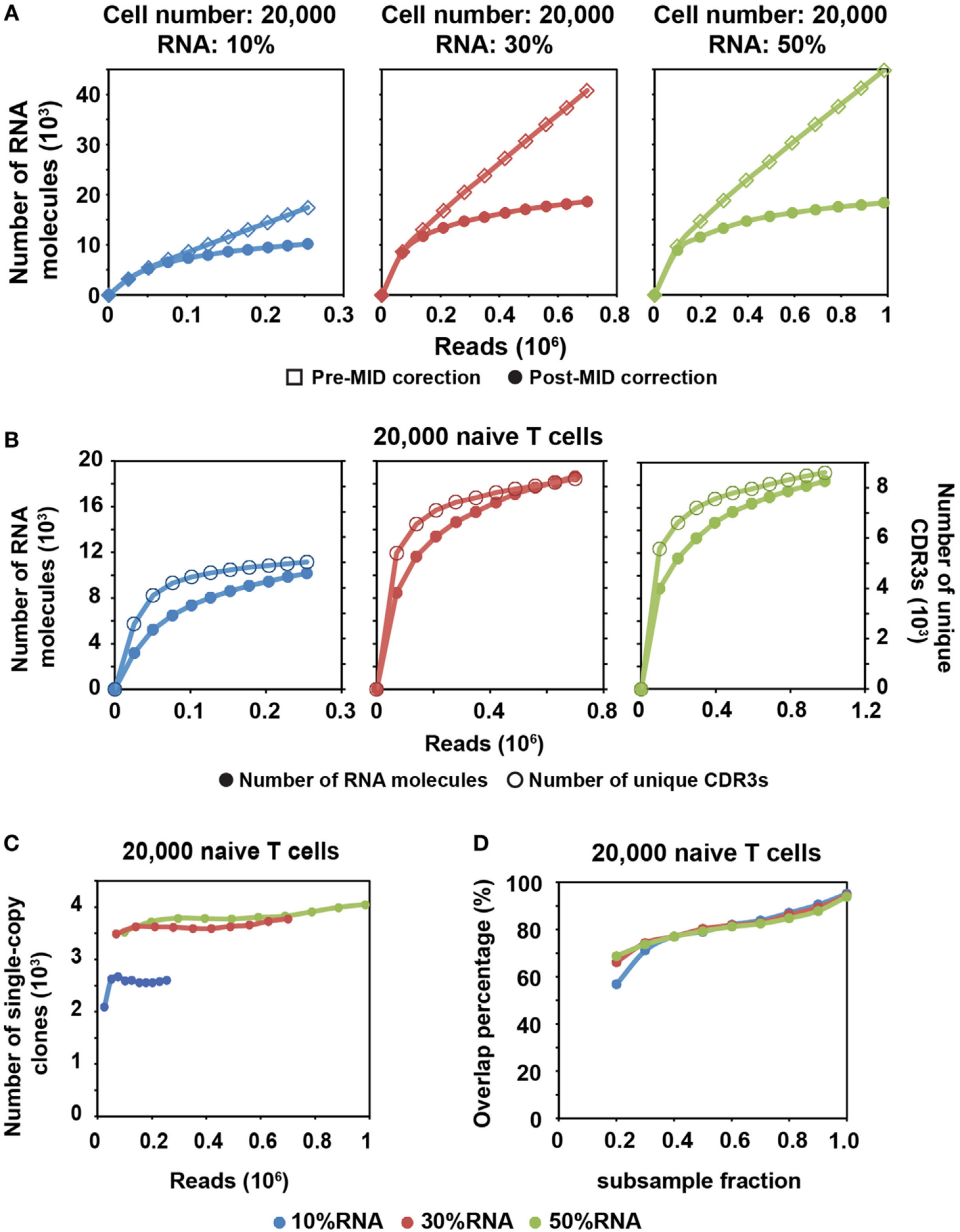
MID Clustering-based IR-Seq is capable of accurate digital counting of T cell receptor (TCR) RNA molecules. **(A)** Rarefaction curve of detected TCR RNA molecules before and after error correction on molecular identifiers (MIDs) in 20,000 naïve CD8^+^ T cells for three RNA input amounts. Data from other cell inputs are in Figure S4 in Supplementary Material. **(B)** Comparison of rarefaction curve of detected RNA molecules and unique complementarity-determining regions 3 (CDR3s) in 20,000 naïve CD8^+^ T cells for three RNA input amounts. **(C)** Rarefaction curve of number of unique CDR3s with single RNA copy in 20,000 naïve CD8^+^ T cells for three RNA input amounts. Sequencing reads were subsampled to different depth and unique CDR3s were tallied. Data from other cell inputs are in Figure S6A in Supplementary Material. **(D)** The percentage of overlapping clones with single RNA copy at different sequencing depths by sub-sampling in 20,000 naïve CD8^+^ T cells for three RNA input amounts. The overlapping clones were compared between two adjacent sub-samplings and overlap percentage was calculated by dividing the number of overlapping clones by the total number of clones observed in the deeper sub-sampling. Data from other cell input are in Figure S6B in Supplementary Material.

Besides correcting PCR and sequencing errors, MIDs have also been used for absolute quantification of RNA molecule copy number in single-cell studies to improve precision (30–33). Here, we demonstrated how to use MIDCIRS TCR-seq to digitally count TCR transcripts. The absolute quantification of TCR transcripts is fundamental for accurate clonal size estimation. We noticed that PCR and sequencing errors also affected MIDs, as seen in single-cell RNA sequencing studies (29, 34), leading to an inflated number of RNA molecules when libraries were sequenced exhaustively with respective to the total TCR transcripts in the sample (Figure 2A; Figure S4 in Supplementary Material). To correct MID errors, we first removed singleton reads, which cannot be confidently used in generating MID groups due to sequencing errors. Then, we adopted a similar approach applied in single-cell RNA-seq by fitting the distribution of reads under each MID subgroup into two negative binomial distributions (Figure S5 in Supplementary Material) (34). Erroneous MIDs generated due to PCR errors generally have distinctively lower read counts compared with true MIDs. These two negative binomial distributions distinctly separated true MIDs from erroneous MIDs. MIDs with low read counts were removed accordingly (see Materials and Methods). After MID correction, number of RNA molecules saturated across libraries (Figure 2A; Figure S4 in Supplementary Material).

We found that a shallower sequencing depth is required to saturate unique CDR3s than RNA molecules (Figure 2B). In addition, the amount of diversity covered increased with increasing RNA input. Thus, to exhaustively measure the TCR repertoire diversity, with 30–50% of RNA input, a sequencing depth equivalent to 10 times the cell number covers most of the CDR3 diversity (Figure 1C; Figure S2 in Supplementary Material), while a sequencing depth equivalent to about 100 times the relative RNA input (defined as cell number multiplied by percentage of RNA input) is required to saturate the RNA molecules (Figure 2A; Figure S4 in Supplementary Material). For example, 30% RNA of 20,000 cells is equivalent to 6,000 RNA input. Then, it takes about 600,000 reads to saturate the RNA molecules but only 200,000 reads to saturate the unique CDR3s (Figure 2A, middle panel).

After MID correction, with optimal sequencing depth, we stably detected TCR clones with a single TCR RNA molecule (single-copy clones with at least two identical sequencing reads). The number of single-copy clones saturates with adequate sequencing depth (Figure 2C; Figure S6A in Supplementary Material). Meanwhile, we compared the degree of overlapping clones within these single-copy clones at different sequencing depths. To do this, we subsampled each library to different fractions of the total reads. The overlapping clones were compared between two adjacent subsamples, and the overlap percentage was calculated by dividing the number of overlapping clones by the total number of clones observed in the deeper subsample. Thus, for total of 10 subsamples, 9 clonal overlap percentages were calculated and plotted with respect to sequencing depth (Figure 2D; Figure S6B in Supplementary Material). More than 90% of single-copy clones were repeatedly detected between the full sequencing reads and the 0.9 subsample fraction. The overlap percentage was above 80% for the latter part of curve (Figure 2D; Figure S6B in Supplementary Material), which suggested that we have reached optimal sequencing depth to detect single-copy TCR clones.

### Estimating TCR RNA Molecule Copy Number and Validation with dPCR

**Figure 3:**
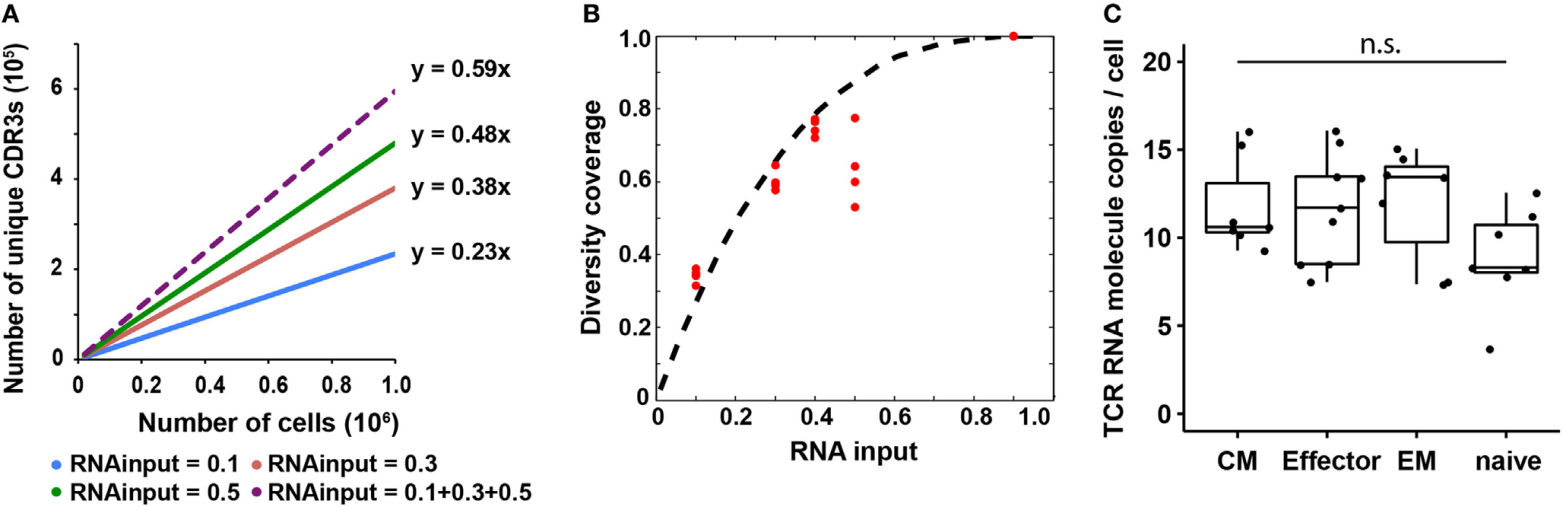
T cell receptor (TCR) RNA copy number per cell estimation and experimental validation. **(A)** Diversity coverage of unique productive complementarity-determining regions 3 with different RNA inputs and cell numbers (Line represents linear regression fit, *F*-test on the slope, *R*^2^ > 0.99 and *p* < 10^−3^ for all different RNA inputs). **(B)** Diversity coverages with different RNA inputs using 3 as a predicted TCR RNA molecule copy number per cell. Dashed line is the theoretical prediction (see Materials and Methods); red dots are diversity coverages observed in libraries with different RNA inputs as illustrated in panel **(A)**, assuming diversity coverage at 90% RNA input is 1. **(C)** Digital PCR results of TCR RNA molecule copies per cell in different CD8^+^ T cell subset (N, naïve; CM, central memory; EM, effector memory; E, effector; NTC, no template control; n.s: *p*-value > 0.05 by Mann–Whitney *U* test).

From early analysis, we know that the diversity coverage of unique CDR3s increased as RNA input increased. Here, we performed an in depth analysis on the relationship between these two parameters and found that the diversity coverage of unique CDR3s increased significantly as the RNA input increased initially, then reached a plateau, which resulted in a nonlinear increasing of the diversity coverage of unique CDR3s (Figures 3A,B). We assumed that total diversity for a sample is the diversity discovered when combining all sequencing reads from 10, 30, and 50% RNA input libraries into a pseudo-90% RNA input. With 50% RNA, we could recover about 60% of total diversity (Figure 3B).

Since the observed diversity is dependent on total TCR RNA molecules in a sample, which is a function of TCR RNA molecule copy number per cell and RNA input percentage, we next sought to use a probability model to predict TCR RNA molecule copy number per cell using the observed diversity coverage of unique CDR3s as a function of RNA input percentage (see Materials and Methods). We used the estimated diversity coverage of different RNA inputs, including 10, 30, and 50% RNA, as well as the computationally combined pseudo-40% (10 + 30%) and pseudo-90% RNA inputs as data points to fit the probability model. The best fit resulted in three copies of TCR RNA molecule per cell (Figure 3B). In another independent experiment, RNA from 20,000 and 100,000 naïve CD8^+^ T cells were evenly separated into five aliquots, respectively. Four of five aliquots were sequenced (Table S2 in Supplementary Material). Results showed that CDR3 diversity detected by MIDCIRS is very reproducible among the four aliquots and is also proportional to the cell input numbers. In addition, we bioinformatically combined the aliquots into pseudo-40, -60, and -80% of RNA inputs and fitted the diversity coverage using the probability model described in the Section “Materials and Methods.” As with previously, the best fit resulted in three copies of TCR RNA molecule per cell (Figure S7 in Supplementary Material).

However, in order to apply this TCR RNA molecule copy number in estimating T cell clone size, we need to validate it using a different method and also test to see if different phenotypes of T cells might have different TCR RNA molecule copy numbers, which would be similar to the differences seeing in naïve B cells and plasmablasts (35). Next, we validated TCR RNA molecule copy number using dPCR and found that various types of T cells have similar TCR RNA copies (8–12 copies per cell) (Figure 3C). Thus, with MIDCIRS TCR-seq, we could achieve about 30% efficiency in recovering the target TCR RNA molecules, which is expected given dPCR in a nanoliter volume is more efficient than bulk PCR in tubes (36). This ratio also establishes a reference point for rare T cell clone frequency estimate using MIDCIRS method.

### Detecting Single-Cell Worth of TCR RNA Using MIDCIRS

**Figure 4:**
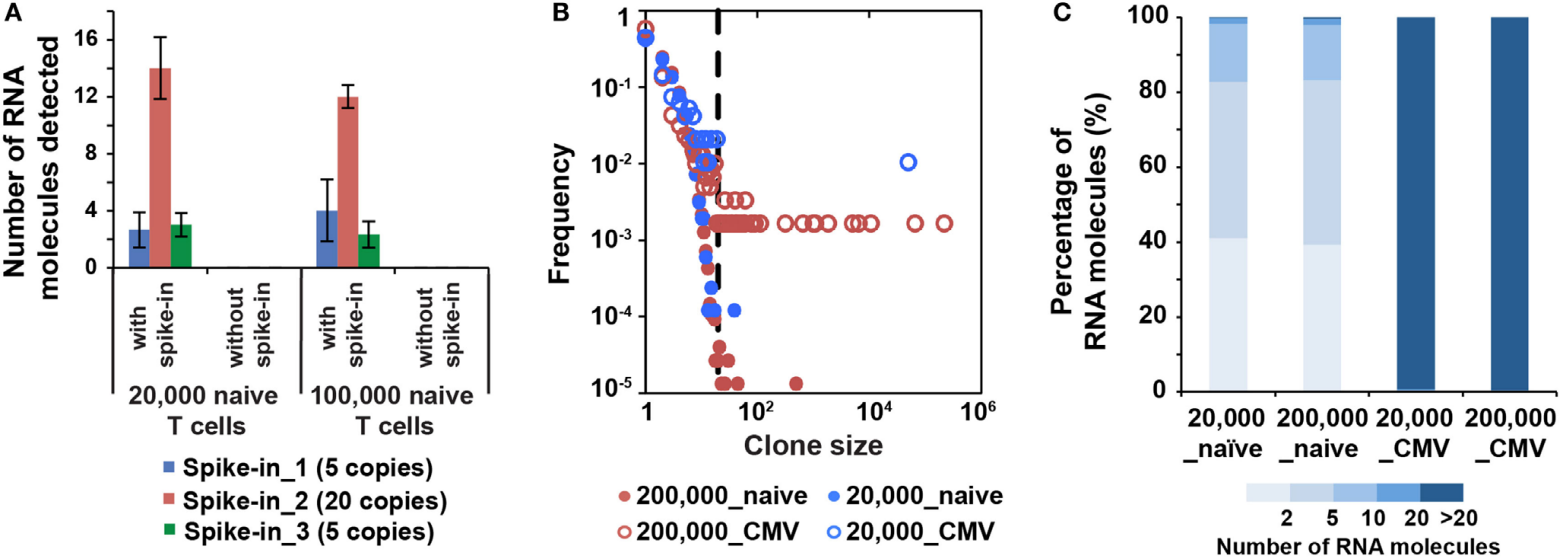
MID Clustering-based IR-Seq is sensitive to detect both low copy and highly clonal expanded T cell receptors (TCRs). **(A)** Number of RNA molecules detected by sequencing for each spike-in TCR control sequences (the numbers in the legend denote copies of each TCR spike-in control sequence added). **(B)** Comparison of clone size distribution in naïve CD8^+^ T cells and CMVpp65-specific effector CD8^+^ T cells (dashed line indicates TCR sequences with 20 copies of RNA molecules). **(C)** The percentage of RNA molecules that varying degree of clonally expanded complementarity-determining region 3 account for.

The lack of accurate and absolute quantitation of TCR clones limited the evaluation of the sensitivity of various IR-seq methods (37), which slowed the application of detecting rare TCR clones in both basic research and clinical practice. To address the detection sensitivity using MIDCIRS, we spiked-in control TCR RNA with varying copy numbers into naïve T cells and validated the robustness of detecting spiked-in TCRs. 5, 20, and 5 copies of three spike-in cell lines with known TCR sequences were added into 20,000 and 100,000 naïve CD8^+^ T cells. 3, 13, and 3 copies of three spike-ins were reliably detected, respectively (Figure 4A).

We also analyzed the ability to detect a single T cell’s worth of control RNA in a larger number of other T cells. We digitally counted the concentration of TCR RNA molecule from the Jurkat cell line and spiked-in 10 copies of TCR RNA into 20,000–1,000,000 naïve CD8^+^ T cells (Table S1 in Supplementary Material). In all 1,000,000 cells we sequenced, we were capable of detecting Jurkat TCR sequences (Table 1). This sensitivity was a significant improvement compared with previous method, which was demonstrated to be 1 in 10,000 (21). These results demonstrated that MIDCIRS is highly sensitive, capable of detecting a single-cell’s amount of TCR transcripts, and rare clones could be readily and robustly detected. Those single-copy clones (minimum two identical reads) we discovered are thus likely to come from single cells (Figure 2C; Figure S6A in Supplementary Material).

**Table 1:** Spike-in Jurkat T cell receptor (TCR) RNA detection in naïve CD8^+^ T cells.

Sample	Jurkat TCR copies detected
20,000Tn_10%RNA	7
20,000Tn_30%RNA	0
20,000Tn_50%RNA	1
100,000Tn_10%RNA	5
100,000Tn_30%RNA	4
100,000Tn_50%RNA	1
200,000Tn_10%RNA	7
200,000Tn_30%RNA	3
200,000Tn_50%RNA	3
1,000,000Tn_10%RNA	4
1,000,000Tn_30%RNA	8
1,000,000Tn_50%RNA	17

*10 TCR-copy worth of Jurkat RNA was added to each sample during the reverse transcription step. Number of molecular identifiers for RNA molecules that are tagged with jurkat TCR sequences were counted*.

Meanwhile, we compared the sensitivity of MIDCIRS and 5′RACE protocol using the diversity coverage as the parameter. Briefly, the 5′RACE protocol that was used in Smart-seq2 protocol was used for TCR-seq, which has been demonstrated to significantly improve RNA capture efficiency (38). Equal amount of RNA (20%) from same purification was used for both MIDCIRS and 5′RACE protocol. We then processed sequencing results with MIDCIRS-TCR pipeline and found that 5′RACE protocol only recovered about 44% of diversity compared to what MIDCIRS protocol obtained (Table S3 in Supplementary Material). With improved accuracy and sensitivity to detect rare clones, MIDCIRS is promising in being applied to detect MRD after treatment.

### Quantifying T Cell Clonal Expansion in Infection Using MIDCIRS

It has been shown that the clonality and quantity of T cells are strongly correlated with efficacy of therapies, such as cancer chemotherapy and antiviral therapy (20, 39). Accurate quantification of diversity and abundance of T cell clones is important for application of TCR-seq in clinical settings, ranging from prognosis to treatment decision-making. However, there lacks an accurate approach to evaluate the degree of T cell clonal expansion in humans. Therefore, we applied MIDCIRS TCR-seq to examine T cell clonal expansion in infection. We sorted 20,000 and 200,000 CMVpp65-specific effector CD8^+^ T cells from CMV-infected patients and used 30% of RNA input to perform TCR-seq (Table S4 in Supplementary Material). CMV pp65 peptide has been shown to be the immunodominant target of CD8^+^ T cell response (40). TCR RNA molecules were digitally counted through MIDCIRS pipeline. We defined TCR sequences with over 20 copies of RNA molecules as expanded clones according to TCR abundance distribution comparing between naïve CD8^+^ T cells and CMV tetramer positive effector CD8^+^ T cells (Figure 4B). Over 99% unique RNA molecules were from these expanded clones in CMVpp65-specific effector CD8^+^ T cells. On the other hand, although we observed uneven clonal distribution in naïve CD8^+^ T cells, these expanded clones only account for less than 1% unique RNA molecules (Figure 4C). Our data showed that in CMV infection, single CMV-specific TCR clone can have about 70,000 T cell progenies in 200,000 polyclonal CMV-specific effector CD8^+^ T cells (Table S4 in Supplementary Material). These polyclonal CMV-specific effector CD8^+^ T cells represent about 2.6% of total CD8^+^ T cells. In addition, our previous study showed that tetramer positive polyclonal CMV precursor cells existed at a frequency of 1 in 100,000 CD8^+^ T cells in CMV seronegative individuals (22). Taking together, these results suggest that single T cell clone can have about 900-fold proliferation in infection in humans. Thus, MIDCIRS can be applied to evaluate clone size and degree of clonal expansion in viral infection.

## Discussion

In this study, we applied the MIDCIRS, recently developed by our group (9), in T cells to demonstrate (1) the necessity of MID sub-clustering to improve accuracy of repertoire diversity estimation; (2) the accuracy of counting TCR RNA molecules *via* MID read-distribution based barcode correction; (3) the sensitivity of detecting a single cell in as many as one million naïve T cells; and (4) the ability to quantify T cell clonal expansion due to infection in CMV-seropositive patients.

Previous MID-based IR-seq methods, such as MIGEC, build TCR consensus sequences by grouping MIDs (17, 41). However, the number of target molecules could vary significantly with different sample inputs, which could be challenging for choosing the appropriate MID length to ensure that each target RNA molecule is uniquely tagged by MID. Longer MIDs are likely to decrease the reverse transcription efficiency (28, 29). Thus, the MIDCIRS method offers a flexible strategy for MID-barcoded IR-seq. In addition, MIGEC triages MIDs with high diversity as ambiguous. We compared TCR diversity discovered using MIDCIRS with that of MIGEC, using MID with at least two reads as the threshold for both approaches (see Materials and Methods) and found that MIGEC led to an underestimated TCR diversity (Figure S8 in Supplementary Material, *p* < 0.001, effect size *r* = 0.62). We demonstrated that using MID-based sub-clustering approach, MIDCIRS could identify new diversities, prevent chimera sequences from being built, and digitally count RNA molecules (Figure 1; Figures S2 and S3 in Supplementary Material). This corrected diversity is highly consistent with cell input numbers.

While MIDs are useful to correct for sequencing errors and PCR errors that occur on TCR sequences, such errors are also likely to show up on MID sequences. Although these errors do not affect TCR diversity estimation, they lead to an overestimation of transcript copies, thus misestimating TCR clone size (Figure 2; Figure S4 in Supplementary Material). We corrected MID errors based on the distribution of MID read counts under MID subgroups. With MID correction, we were able to accurately count TCR RNA molecule copy number, estimate MIDCIRS detection limit as well as detect T cell clonal expansion.

Noteworthy, we found uneven CDR3 clone size distribution in naïve CD8^+^ T cells (Figure 4B). The most expanded clone was enriched about 0.27% (Table S1 in Supplementary Material). This could be due to convergent recombination as has been previously noted (42, 43) or uneven clonal expansion during thymocyte maturation and selection in thymus (44, 45).

Furthermore, there is a lack of standard guidelines of how much RNA input to use for library preparation and sequencing. Also, the capacity to evaluate immune repertoire and gene expression profile simultaneously will facilitate clinical practice, such as cancer immunotherapies. Efforts have been made to reconstruct antibody and TCR repertoire from RNA-seq data. This, however, requires very deep sequencing to recover highly expanded T cell clones in the sample, and the exact degree of repertoire coverage is difficult to assess (46–48). Here, we demonstrated that 50% RNA is enough to cover about 60% of CDR3 diversity (Figure 3B), making it beneficial to take advantage of the rest of the RNA from the same sample for other applications, e.g., RNA-seq.

Based on the TCR diversity estimation and its dependency on RNA input, we built a probability model to estimate TCR RNA molecule copies, which resulted in three copies per cell (Figure 3B). We would like to point out that this does not mean that on average there are three copies of TCR RNA in a T cell. Because of the efficiency of RNA purification and reverse transcription, we expect our observed RNA molecule per cell to be lower than the true value. In Fact, dPCR results showed an average of 10 copies of TCR RNA molecule per cell (Figure 3C), suggesting the efficiency of MIDCIRS in TCR RNA molecule digital counting is about 30%, which is consistent with previous finding that nanoliter reaction volume significantly improved PCR efficiency. Thus, quantifying TCR RNA molecule per cell enables us to estimate the extent of T cell clonal expansion that was not possible until now.

We also used spike-in TCR RNA to validate the sensitivity of MIDCIRS. We showed that spiked-in TCR RNA at as few as five copies can be reliably detected across multiple libraries (Figure 4A). More importantly, we were also able to detect a single-cell worth of RNA in as many as one million cells (Table 1). With this demonstrated sensitivity, this method could be extremely useful in MRD detection.

Last, we applied MIDCIRS to evaluate T cell clonal expansion in CMV-infected patients. Through accurate digital counting of TCR RNA molecules and in combination of precursor T cell frequency, we showed that CMV-specific effector CD8^+^ T cells can expand at least 900 times, and there could be more than 70,000 effector CD8^+^ T cells derived from the same CMV-specific T cell clone in total of 7,700,000 of CD8^+^ T cell in infection. We also noticed that there is a potential of same TCR sequences tagged with same MID, which would under estimate the clonal size, especially in highly expanded clones. We calculated the expected number of collisions where same MIDs tag same RNA molecules (Supplementary Methods in Supplementary Material). With MID length being 12, when there are 200,000 identical RNA molecules, the percentage of identical RNA molecules tagged with same MID is only 1%. While long MID decreases the percentage of identical RNA molecules tagged with same MID, it also decreases efficiency of reverse transcription. Our analysis revealed that MID with 12 nucleotides is appropriate. Therefore, MIDCIRS provides the foundation of accurate assessment of clone size and clonal expansion in infection and vaccination, which would be a useful technology to provide a comprehensive quantification of the T cell repertoire in various basic studies and clinical settings.

## Ethics Statement

The protocol of using de-identified blood donors’ sample was approved by the IRB board of University of Texas at Austin.

## Data Access

All sequencing data are under SRA accession SRP128082.

## Author Contributions

K-YM performed all library preparation, data analysis, and wrote the manuscript; CH developed MIDCIRS-TCR analysis pipeline and RNA copy number simulation model; BW helped with naïve T cell sorting and manuscript editing; CW helped with CMV-specific T cell sorting and CMV-specific T cell line culture; JX helped to optimize MIDCIRS pipeline. HY helped with sequencing. NJ conceived the idea, designed the study, directed data analysis, and revised the manuscript with contributions from all coauthors.

## Disclaimer

The protocol of using de-identified blood donors’ sample was approved by the IRB board of University of Texas at Austin.

## Conflict of Interest Statement

NJ is a scientific advisor of ImmuDX, LLC. A provisional patent application has been filed by the University of Texas at Austin on the method described here.
