# Supplementary material for: Immune Repertoire Sequencing Using Molecular Identifiers Enables Accurate Clonality Discovery and Clone Size Quantification
Source: Front Immunol. 2018 Feb 5;9:33. doi: 10.3389/fimmu.2018.00033 (PMC5808239; doi:10.3389/fimmu.2018.00033)
Supplement: Supplementary file 1 [file data_sheet_1.PDF]

Supplementary Material

Immune Repertoire Sequencing using Molecular Identifiers Enables Accurate Clonality Discovery and Clone Size Quantification

Ke-Yue Ma[1#], Chenfeng He[2#], Ben S. Wendel[3], Chad M. Williams[2], Jun Xiao[4], Hui Yang[5,6], Ning Jiang[1,2,*]

[1]Institute for Cellular and Molecular Biology, College of Natural Sciences, The University of Texas at Austin, Austin, Texas, USA.

[2]Department of Biomedical engineering, Cockrell School of Engineering, The University of Texas at Austin, Austin, Texas, USA.

[3]McKetta Department of Chemical Engineering, Cockrell School of Engineering, The University of Texas at Austin, Austin, Texas, USA.

[4]ImmuDX, LLC, Austin, Texas, USA.

[5]School of Life Sciences, Northwestern Polytechnical University, Xi'an, Shaanxi, China

[6]Research Center of Special Environmental Biomechanics & Medical Engineering, Xi'an Shaanxi, China

[#]These authors contributed equally to this work

[*]Corresponding author

Correspondence:

Ning Jiang, Ph.D.

jiang@austin.utexas.edu

Supplementary Methods

Expected number of identical RNA molecules tagged with same MID.

When there are N different MIDs, the probability of RNA molecule B's MID shares RNA molecule A's MID is 1/N. Let the number of identical RNA molecules be n, then the probability that RNA molecule A's MID is shared is:

$$1 - \left(1 - \frac{1}{N}\right)^{n-1} \tag{1}$$

Based on equation (1), the expected number of identical RNA molecules tagged with same MID, *E*(n) is:

$$E(n) = n \times \left(1 - \left(1 - \frac{1}{N}\right)^{n-1}\right) \tag{2}$$

Supplementary Figures and Tables

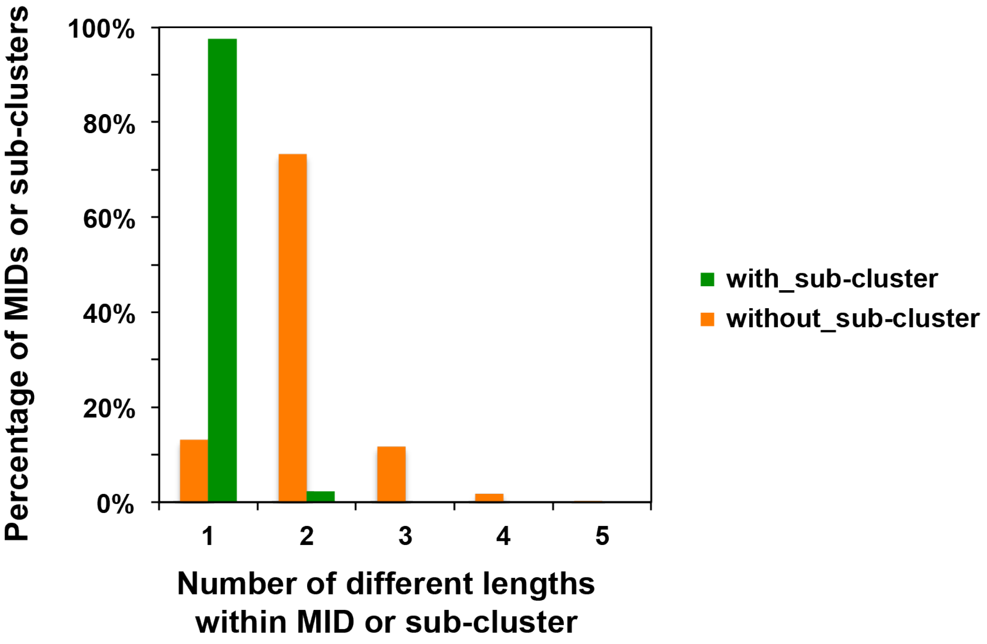

Supplementary Figure S1. CDR3 length differences within multi-RNA containing MIDs before and after sub-clustering. The number of different CDR3 lengths within multi-RNA containing MIDs from one million naïve $CD8^+$ T cells (50% RNA input) was plotted before sub-clustering (orange) and within the sub-clusters (green).

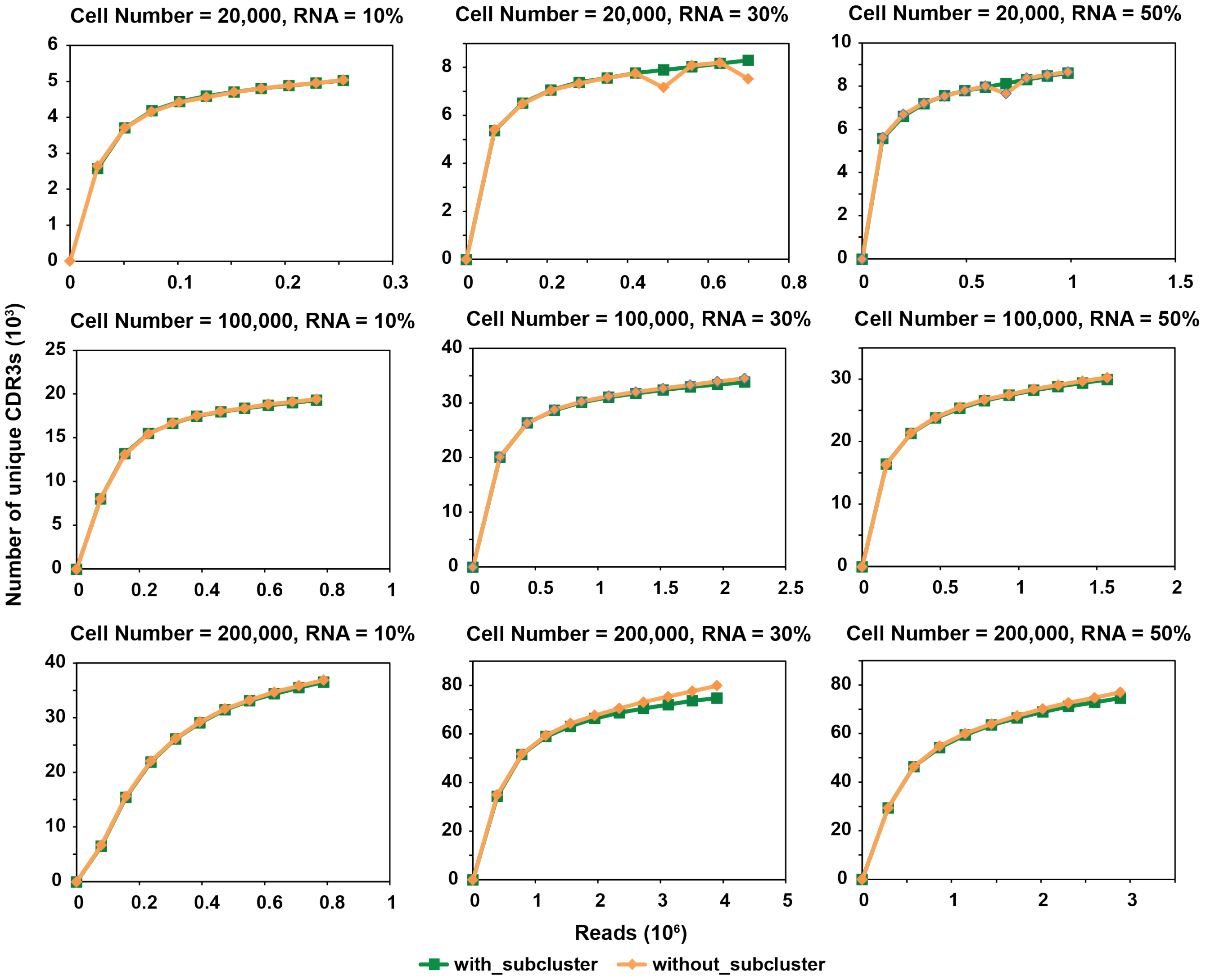

Supplementary Figure S2. Rarefaction curve of unique CDR3s with or without sub-clustering. Number of unique CDR3s in libraries made using three different RNA inputs (10%, 30% and 50%) from sorted 20,000, 100,000 and 200,000 naïve CD8$^+$ T cells are shown here.

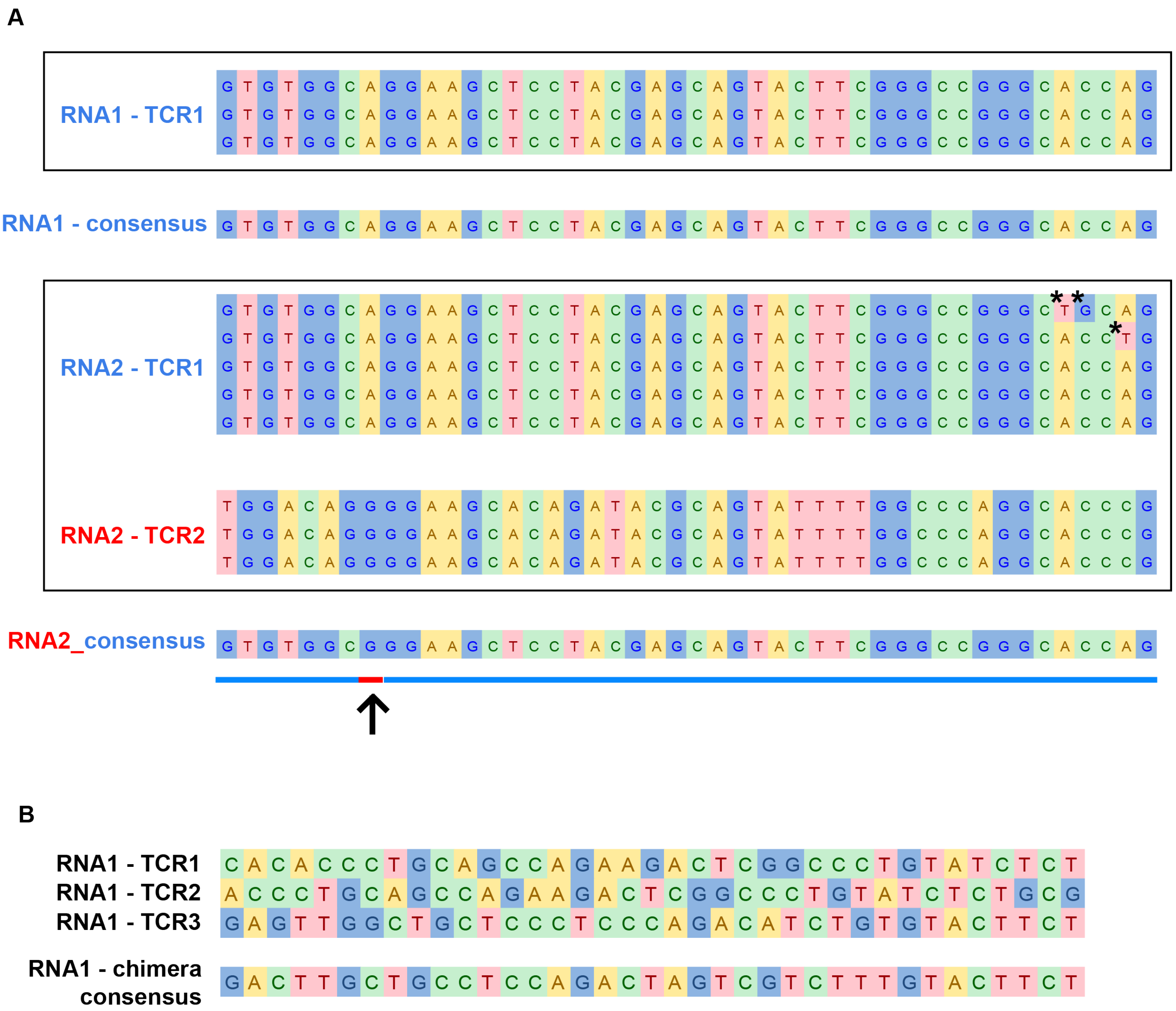

Supplementary Figure S3. Representative demonstration of chimera consensus sequences generated without sub-clustering (chimera TCR sequence in Figure 1C). (A). Two different TCR RNAs (RNA2-TCR1 and RNA2-TCR2) were tagged with the same MID (RNA2), while one of the TCRs (TCR1) has a sister RNA tagged by another MID (RNA1). After building consensus sequence weighted by quality score and number of reads at each nucleotide position, a chimera consensus sequence was generated from RNA2-tagged TCR sequences (Top box, TCR1 tagged with RNA1; bottom box, two TCR sequences tagged with same MID; *, sequencing or PCR errors that are removed in the consensus building; sequence outside the top box, true TCR1 consensus sequence; sequence outside the bottom box, chimera consensus sequence; arrow, chimera nucleotide base that differs from the rest of consensus sequence was generated by weighing read number and quality score at each nucleotide). (B) Multiple singleton TCR RNAs were tagged with the same MID (RNA1) that were generated by either sequencing

or PCR errors. Without sub-clustering, these singletons failed to be removed and a chimera consensus sequence was generated.

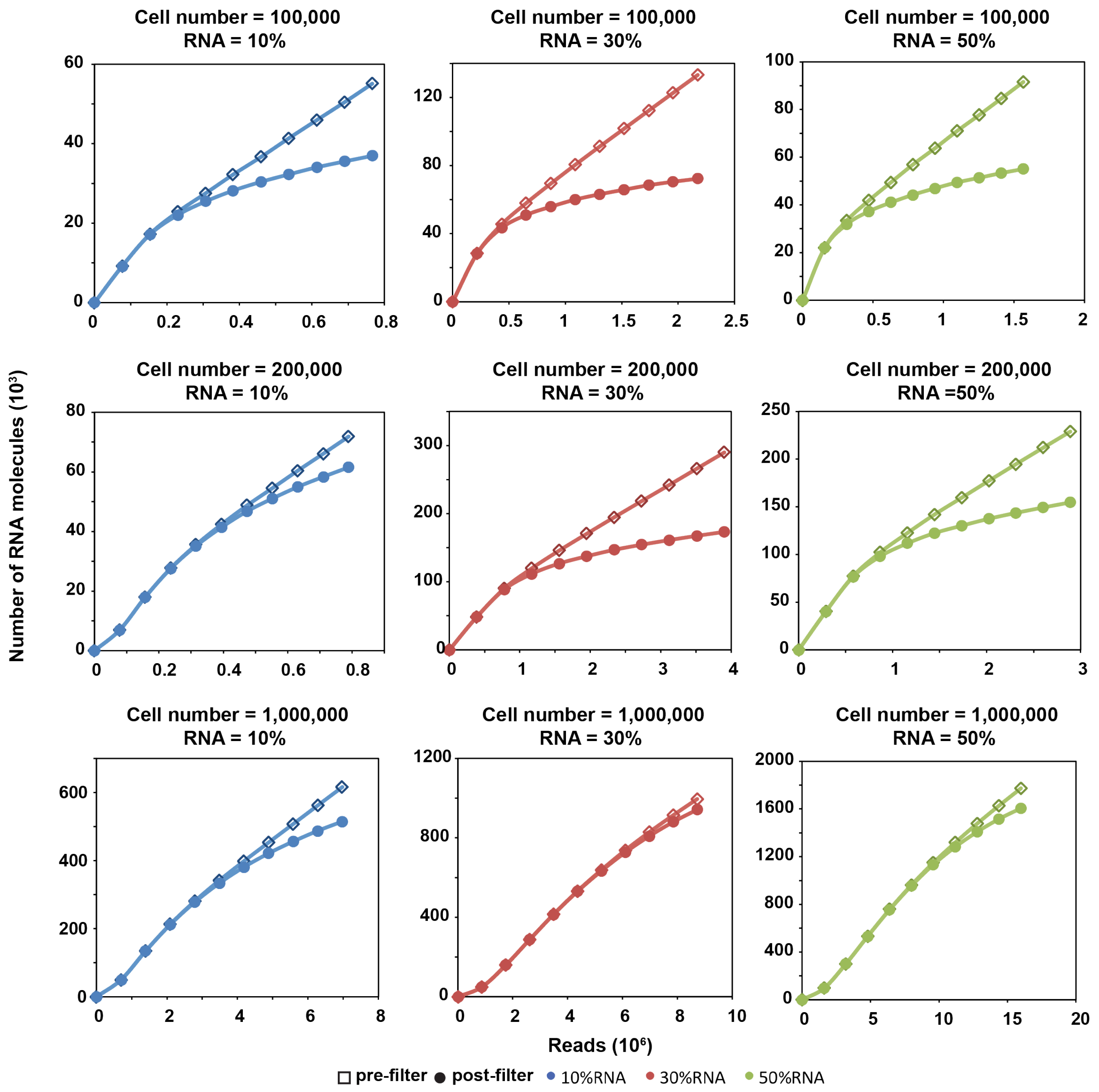

Supplementary Figure S4. Rarefaction curve of detected TCR RNA molecules before and after MID correction in 100,000, 200,000 and 1,000,000 naïve $CD8^+$ T cells for three RNA input amounts.

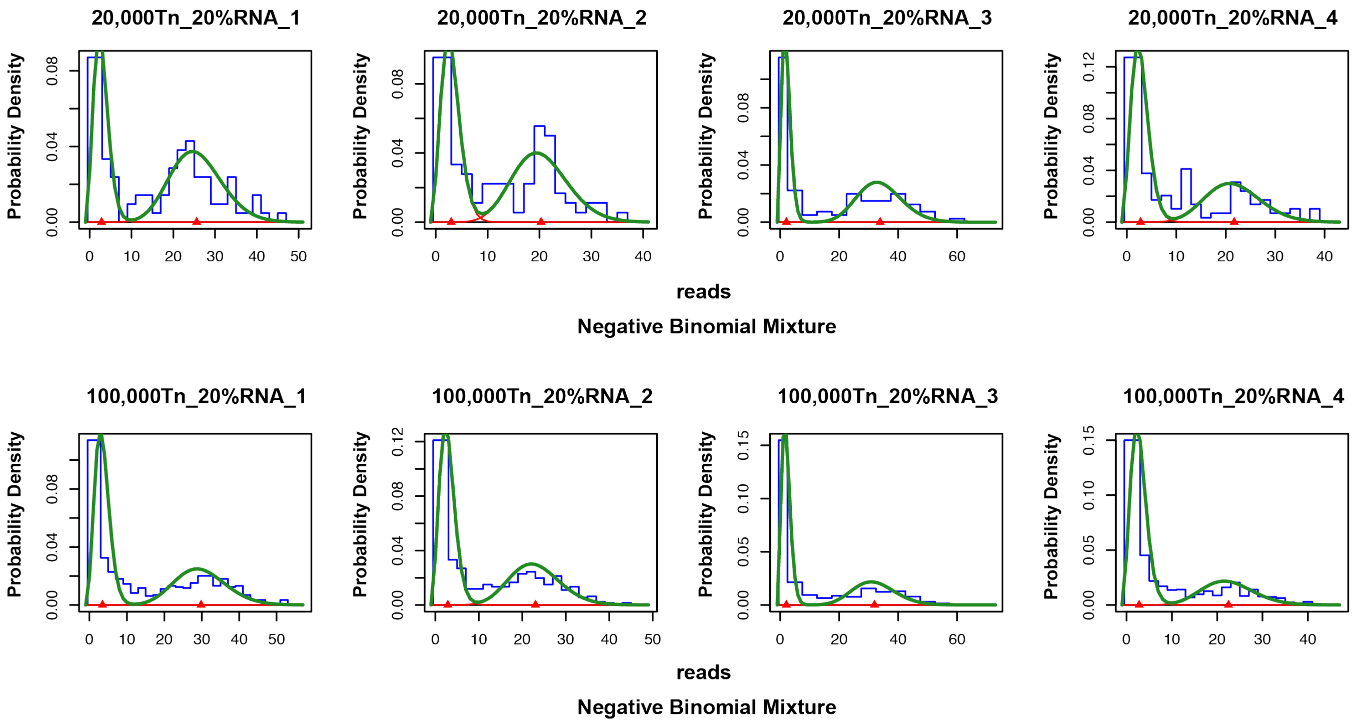

Supplementary Figure S5. Distribution of reads under each MID sub-group. Top expressed unique CDR3 in eight naïve CD8$^+$ T cell libraries were first separated into MID sub-groups, then the histograms of read numbers under each MID sub-group were plotted here (Blue line) (Green line is the final fitting of two negative binomial distributions of the blue line; red line is the fitting of individual negative binomial distributions).

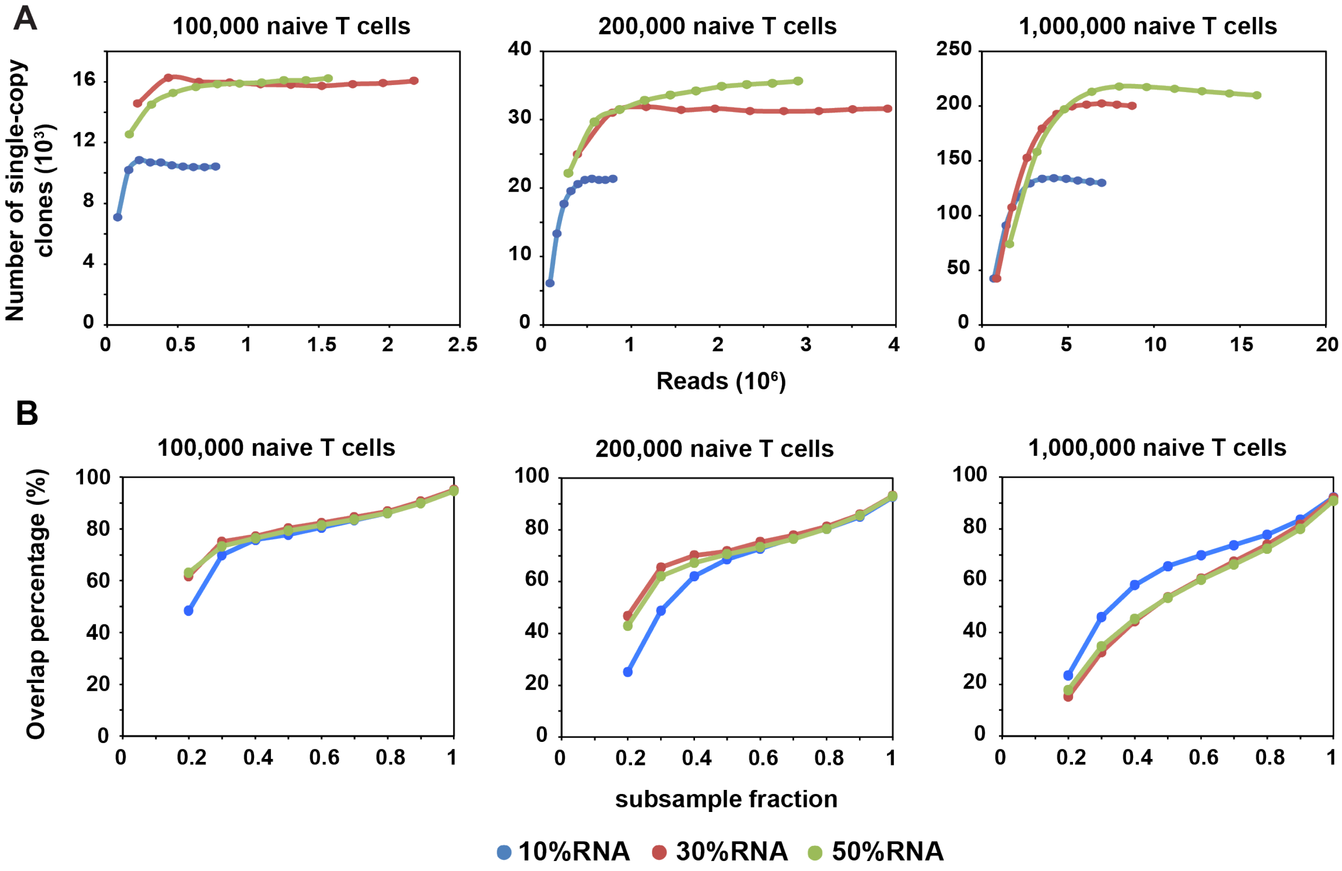

Supplementary Figure S6. MIDCIRS is capable of accurate digital counting of TCR RNA molecules. (A) Rarefaction curve of number of unique CDR3s with single-copy RNA in 100,000, 200,000 and 1,000,000 naïve $CD8^+$ T cells for three RNA input amounts. (B) The percentage of overlapping clones with single-copy of transcript at different sequencing depths by sub-sampling in 100,000, 200,000 and 1,000,000 naïve $CD8^+$ T cells for three RNA input amounts. The overlapping clones were compared between two adjacent sub-samplings and the overlap percentage was calculated by dividing the number of overlapping clones by the total number of clones observed in the deeper sub-sampling.

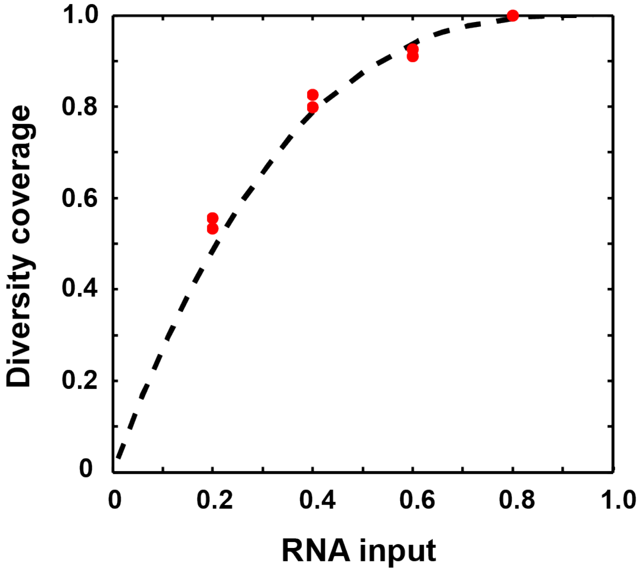

Supplementary Fig. S7. Curve fitting of diversity coverages as a function of different RNA inputs using 3 as a predicted TCR RNA molecule copy number per cell. Dashed line is the theoretical prediction (See **methods**); red dots are diversity coverages observed in libraries with different RNA inputs (20%, pseudo-40%, pseudo-60% and pseudo-80%), assuming diversity coverage at pseudo-80% RNA input is 1.

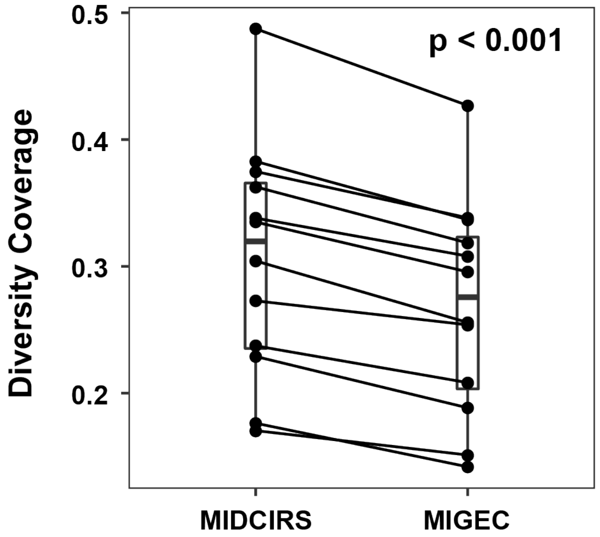

Supplementary Fig. S8. Comparison of diversity coverage between MIDCIRS and MIGEC pipelines on the same set of data presented in this study. P-value was determined by paired Wilcoxon test.

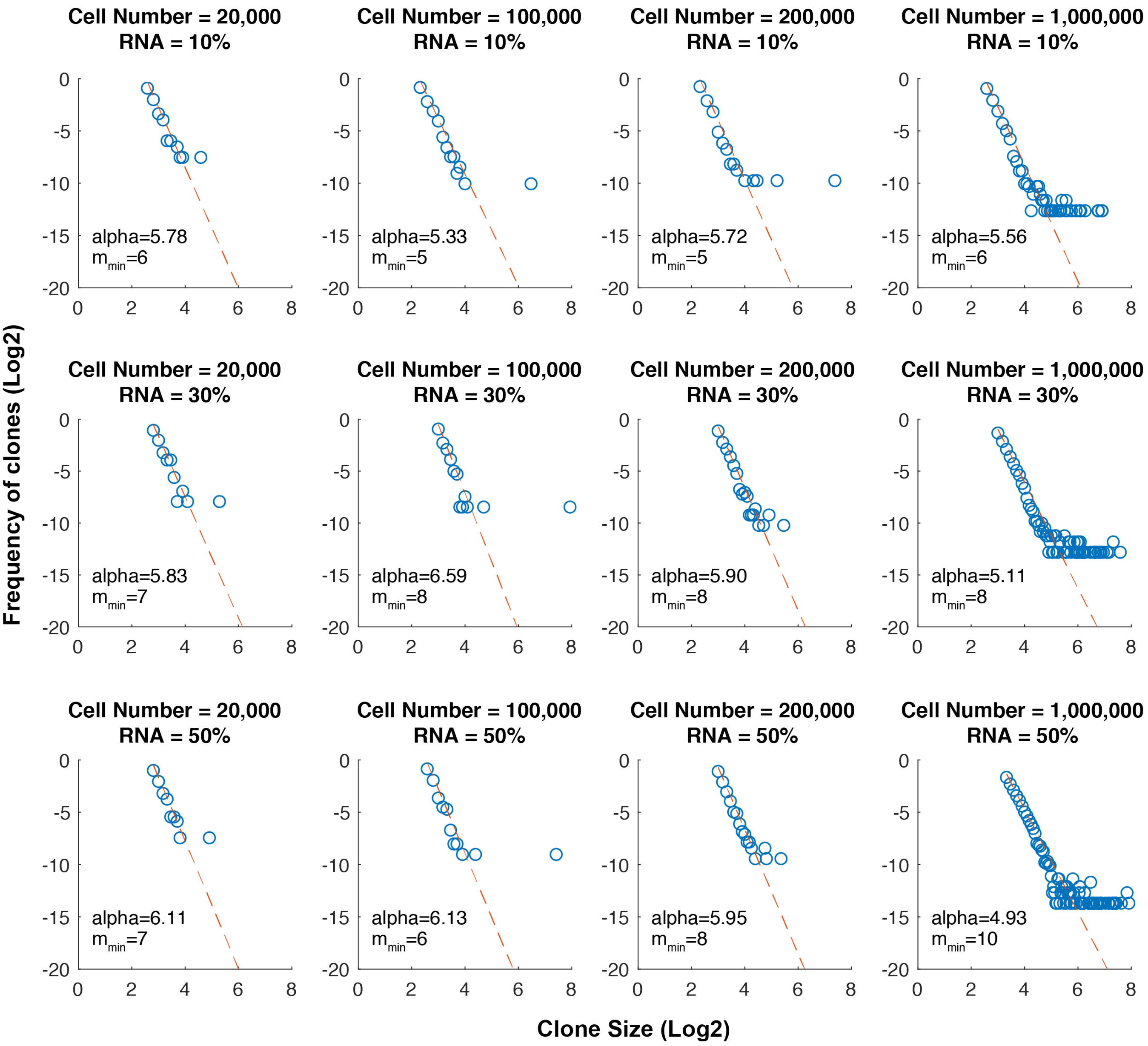

Supplementary Fig. S9. CDR3 clone size distribution of 20,000, 100,000, 200,000 and 1,000,000 naïve CD8$^+$ T cells. Red dashed line is the fitted power law distribution (**See methods**).

Supplementary Table S1. Metrics of sequencing results of first naïve CD8$^+$ T cell experiment.

Sample	Raw reads	Mappable reads	Map percentage (%)	Total RNA molecules	Unique productive CDR3	Percentage of MIDs with sub-clusters (%)	Percentage of chimera sequences (%)	Top CDR3 molecules *	Top CDR3 molecule fraction (%)
20,000Tn _10%RNA	402975	254228	63.09	10171	4579	0.11	0.32	24	0.24
20,000Tn _30%RNA	877556	698961	79.65	18670	7253	0.34	0.42	39	0.21
20,000Tn _50%RNA	1188083	984951	82.90	18367	7495	0.32	0.70	30	0.16
100,000Tn _10%RNA	922615	766441	83.07	36949	17632	0.28	0.33	89	0.24
100,000Tn _30%RNA	2409732	2173270	90.19	72257	30428	0.70	1.58	245	0.34
100,000Tn _50%RNA	1744861	1566048	89.75	55058	27280	0.52	0.99	171	0.31
200,000Tn _10%RNA	1000937	788947	78.82	61525	34097	0.41	0.86	166	0.27
200,000Tn _30%RNA	4224183	3902130	92.38	173224	66990	1.57	5.44	498	0.29
200,000Tn _50%RNA	3147293	2889513	91.81	154666	67607	1.28	2.64	628	0.41
1,000,000Tn _10%RNA	7695858	6975703	90.64	514916	237331	3.19	16.14	1430	0.28
1,000,000Tn _30%RNA	9439612	8719649	92.37	942010	382743	5.18	17.02	2387	0.25
1,000,000Tn _50%RNA	17021339	15979187	93.88	1606258	487295	8.52	47.45	4468	0.28

- Top CDR3: CDR3 with highest MID.

Supplementary Table S2: Metrics of sequencing results of second naïve $CD8^+$ T cell experiment.

Sample	Raw reads	Mappable reads	Map percentage (%)	Total RNA molecules	Unique productiv eCDR3
20,000Tn_20%	334713	293943	87.82	13411	7466
20,000Tn_20%	310547	262774	84.62	13329	7464
20,000Tn_20%	526435	434432	82.52	16873	8888
20,000Tn_20%	447301	360520	80.60	18573	8750
100,000Tn_20%	1962817	1853561	94.43	94536	46272
100,000Tn_20%	1575993	1481210	93.99	87887	44296
100,000Tn_20%	1911879	1776146	92.90	95167	46087
100,000Tn_20%	1858400	1721522	92.63	114885	48601

Supplementary Table S3: Metrics of sequencing results of naïve CD8$^+$ T cell with MIDICRS and 5'RACE.

Sample	Protocol	Raw reads	Mappable reads	Map percentage (%)	Unique productive CDR3	Ratio on unique CDR3 discovered (MIDCIRS/5'RACE)
20,000Tn_20%RNA_1	MIDCIRS	56780	46809	82.44	4202	2.77
	5'RACE	74603	55268	74.08	1516	
20,000Tn_20%RNA_2	MIDCIRS	53322	42036	78.83	4284	2.42
	5'RACE	77696	61074	78.61	1767	
100,000Tn_20%RNA	MIDCIRS	432015	396472	91.77	28975	2.15
	5'RACE	406533	336487	82.77	13497	
200,000Tn_20%RNA_1	MIDCIRS	815238	758556	93.05	55052	1.92
	5'RACE	885269	734108	82.92	28705	
200,000Tn_20%RNA_2	MIDCIRS	812503	649791	79.97	51870	2.03
	5'RACE	813019	674146	82.92	25548	

Supplementary Table S4: Metrics of sequencing results of CMV-specific effector $CD8^{+}$ T cell experiments.

Sample	Mappable reads	Total RNA molecules	Unique productive CDR3	Top CDR3 molecules	Top T cell clone size (*)
200000 Teffector_30%RNA	2655814	324238	423	216348	72116
20000 Teffector_30%RNA	293931	40815	88	40532	13510

(*): Assuming 3 copies of RNA are recovered per cell according to figure 4.

Supplementary Table S5: MIDCIRS and digital PCR primers used in this paper.

**Reverse transcription primer:**	
RT	ACACTCTTTCCCTACACGACGCTCTTCCGATCT NNNNNNNNNNNN GACCTCGGGTGGGAACAC (N indicates random molecular barcode)
**1st PCR primers:**	
1st PCR reverse	ACACTCTTTCCCTACACGAC
1st PCR forward:	
TRBV1	GACGTGTGCTCTTCCGATCTCTGACAGCTCTCGCTTATACCTTCA
TRBV2	GACGTGTGCTCTTCCGATCTGCCTGATGGATCAAATTTCACTCTG
TRBV3	GACGTGTGCTCTTCCGATCTAATGAAACAGTTCCAAATCGMTTCT
TRBV4	GACGTGTGCTCTTCCGATCTCCAAGTCGCTTCTCACCTGAAT
TRBV5-1	GACGTGTGCTCTTCCGATCTCGCCAGTTCTCTAACTCTCGCTCT
TRBV5-2	GACGTGTGCTCTTCCGATCTTTACTGAGTCAAACACGGAGCTAGG
TRBV5-3	GACGTGTGCTCTTCCGATCTCTCTGAGATGAATGTGAGTGCCTTG
TRBV5-4/5/6/7/8	GACGTGTGCTCTTCCGATCTCTGAGCTGAATGTGAACGCCTTG
TRBV6-1	GACGTGTGCTCTTCCGATCTTCTCCAGATTAAACAAACGGGAGTT
TRBV6-2/3	GACGTGTGCTCTTCCGATCTCTGATGGCTACAATGTCTCCAGATT
TRBV6-4	GACGTGTGCTCTTCCGATCTAGTGTCTCCAGAGCAAACACAGATG
TRBV6-5/6/7	GACGTGTGCTCTTCCGATCTGTCTCCAGATCAAMCACAGAGGATT
TRBV6-8/9	GACGTGTGCTCTTCCGATCTAAACACAGAGGATTTCCCRCTCAG
TRBV7-1	GACGTGTGCTCTTCCGATCTGTCTGAGGGATCCATCTCCACTC
TRBV7-2	GACGTGTGCTCTTCCGATCTTCGCTTCTCTGCAGAGAGGACTGG
TRBV7-3	GACGTGTGCTCTTCCGATCTCTGAGGGATCCGTCTCTACTCTGAA
TRBV7-4/8	GACGTGTGCTCTTCCGATCTCTGAGRGATCCGTCTCCACTCTG
TRBV7-5	GACGTGTGCTCTTCCGATCTGGTCTGAGGATCTTTCTCCACCT
TRBV7-6/7	GACGTGTGCTCTTCCGATCTGAGGGATCCATCTCCACTCTGAC
TRBV7-9	GACGTGTGCTCTTCCGATCTCTGCAGAGAGGCCTAAGGGATCT
TRBV8-1	GACGTGTGCTCTTCCGATCTAAGCTCAAGCATTTTCCCTCAAC
TRBV8-2	GACGTGTGCTCTTCCGATCTATGTCACAGAGGGGTACTGTGTTTC
TRBV9	GACGTGTGCTCTTCCGATCTACAGTTCCCTGACTTGCACTCTG
TRBV10-1/3	GACGTGTGCTCTTCCGATCTACAAAGGAGAAGTCTCAGATGGCTA
TRBV10-2	GACGTGTGCTCTTCCGATCTTGTCTCCAGATCCAAGACAGAGAA
TRBV11	GACGTGTGCTCTTCCGATCTCTGCAGAGAGGCTCAAAGGAGTAG
TRBV12-1/2	GACGTGTGCTCTTCCGATCTATCATTCTCYACTCTGAGGATCCAR
TRVB12-3/4/5	GACGTGTGCTCTTCCGATCTACTCTGARGATCCAGCCCTCAGAAC
TRBV13	GACGTGTGCTCTTCCGATCTCAGCTCAACAGTTCAGTGACTATCAT
TRBV14	GACGTGTGCTCTTCCGATCTGAAAGGACTGGAGGGACGTATTCTA
TRBV15	GACGTGTGCTCTTCCGATCTGCCGAACACTTCTTTCTGCTTTCT
TRBV16	GACGTGTGCTCTTCCGATCTATTTTCAGCTAAGTGCCTCCCAAAT
TRBV17	GACGTGTGCTCTTCCGATCTCACAGCTGAAAGACCTAACGGAAC
TRBV18	GACGTGTGCTCTTCCGATCTATTTTCTGCTGAATTTCCCAAAGAG
TRBV19	GACGTGTGCTCTTCCGATCTGTCTCTCGGGAGAAGAAGGAATC
TRBV20-1	GACGTGTGCTCTTCCGATCTGACAAGTTTCTCATCAACCATGCAA

TRBV21-1	GACGTGTGCTCTTCCGATCTCAATGCTCCAAAAACTCATCCTGT
TRBV22-1	GACGTGTGCTCTTCCGATCTAGGAGAAGGGGCTATTTCTTCTCAG
TRBV23-1	GACGTGTGCTCTTCCGATCTATTCTCATCTCAATGCCCCAAGAAC
TRBV24-1	GACGTGTGCTCTTCCGATCTGACAGGCACAGGCTAAATTCTCC
TRBV25-1	GACGTGTGCTCTTCCGATCTAGTCTCCAGAATAAGGACGGAGCAT
TRBV26	GACGTGTGCTCTTCCGATCTCTCTGAGGGGTATCATGTTTCTTGA
TRBV27	GACGTGTGCTCTTCCGATCTCAAAGTCTCTCGAAAAGAGAAGAGGA
TRBV28	GACGTGTGCTCTTCCGATCTAAGAAGGAGCGCTTCTCCCTGATT
TRBV29-1	GACGTGTGCTCTTCCGATCTCGCCCAAACCTAACATTCTCAA
TRBV30	GACGTGTGCTCTTCCGATCTCCAGAATCTCTCAGCCTCCAGAC
**2nd PCR primers**	
2nd PCR reverse	AATGATACGGCGACCACCGAGATCTACACTCTTTCCCTACACGAC
2nd PCR forward	CAAGCAGAAGACGGCATACGAGATAA XXXXXX GTGACTGGAGTTCAGACGTGTGCTCTTCCGATCT (X indicates fixed library index)
**Digital PCR primers:**	
RT	TTTTTTTTTTTTTTTTTTTTTTTTVN
TRBC_F	GAGCCATCAGAAGCAGAGATC
TRBC_R	CTCCTTCCCATTCACCCAC
TRBC_Probe	CCACACCCAAAAGGCCACACTG
